# Next-Generation Sequencing Revealed a Distinct Immunoglobulin Repertoire with Specific Mutation Hotspots in Acute Myeloid Leukemia

**DOI:** 10.3390/biology11020161

**Published:** 2022-01-19

**Authors:** Miaoran Xia, Lina Wu, Xiaoping Sun, Xin Han, Huige Yan, Jing Huang, Youhui Zhang, Zhihong Hu, Youli Zu, C. Cameron Yin, Xiaoyan Qiu

**Affiliations:** 1 Department of Immunology, School of Basic Medical Science, Peking University, Beijing 100191, China; mxia@ccmu.edu.cn (M.X.); yhg@pku.edu.cn (H.Y.); huangjing82@bjmu.edu.cn (J.H.); 2 Department of Hematopathology, The University of Texas MD Anderson Cancer Center, Houston, TX 77030, USA; lnwu@bjmu.edu.cn (L.W.); zhihong.hu@uth.tmc.edu (Z.H.); 3 Department of Immunology, School of Basic Medical Science, Capital Medical University, Beijing 100069, China; 4 Central Laboratory, Key Laboratory of Carcinogenesis and Translational Research (Ministry of Education/Beijing), Peking University Cancer Hospital & Institute, Beijing 100142, China; 5 Department of Laboratory Medicine, The University of Texas MD Anderson Cancer Center, Houston, TX 77030, USA; xsun@mdanderson.org (X.S.); xhan@mdanderson.org (X.H.); 6 Department of Immunology, Cancer Institute & Hospital, Chinese Academy of Medical Science, Beijing 100021, China; zhyhch@126.com; 7 Department of Pathology & Laboratory Medicine, The University of Texas Health Science Center, Houston, TX 77030, USA; 8 Department of Pathology and Genomic Medicine, Houston Methodist Hospital, Houston, TX 77030, USA

**Keywords:** acute myeloid leukemia, Ig, next-generation sequencing, V(D)J rearrangement, somatic hypermutation

## Abstract

**Simple Summary:**

Identifying new molecular targets is of great importance for prognosis prediction and target therapy of acute myeloid leukemia (AML). We previously reported on frequent expression of immunoglobulin (Ig) in myeloblasts. In this study, we investigated the clinical significance of Ig expression in sorted myeloblasts from 59 AML patients. We found that a higher level of AML-derived Ig expression correlated with a significantly shorter disease-free survival. Furthermore, we performed a comprehensive analysis of AML-derived Ig repertoire by next-generation sequencing (NGS) in 16 patients. The transcripts of AML-derived Ig shared some features with B cell-derived Ig, such as a typical V(D)J recombination and high mutation rates. However, they also showed distinct features. In contrast to the huge diversity of classical Ig, the V_H_-D-J_H_ rearrangements used by AML-derived Ig were biased in each AML patient. In particularly, the Vκ-Jκ rearrangements were skewed in both AML blasts and normal peripheral blood mononucleated cells (PBMCs). However, AML-derived IGK showed high somatic mutation rates (>2%), while IGK in normal PBMCs rarely displayed hypermutation (<2%). More importantly, we identified five mutation hotspots at serine codons of IGKV3-20 in AML blasts, which may be involved in leukemogenesis and serve as a novel marker for disease monitoring and target therapy.

**Abstract:**

Immunoglobulin (Ig) is known as a hallmark of B-lymphocytes exerting antibody functions. However, our previous studies demonstrated that myeloblasts from acute myeloid leukemia (AML) patients could also express Ig with distinct roles. Here, we quantified Ig (IGHG and IGK) transcripts by real-time PCR and performed a comprehensive analysis of Ig repertoire (both heavy chains and light chains) in AML blasts. We found that Ig was frequently expressed by AML blasts. A higher level of AML-derived IGHG expression correlated with a significantly shorter disease-free survival. Next-generation sequencing revealed dysregulated transcripts of all five Ig classes (IGHA, IGHD, IGHE, IGHG, and IGHM) and two Ig types (IGK and IGL) in AML. V_H_-D-J_H_ rearrangements in myeloblasts were biased with individual specificity rather than generally diverse as in B-cells. Compared to AML-derived IgH, AML-derived IGK was more conserved among different AML samples. The frequently shared Vκ-Jκ patterns were IGKV3-20*01/IGKJ1*01, IGKV2D-28*01/IGKJ1*01, and IGKV4-1*01/IGKJ1*01. Moreover, AML-derived IGK was different from classical IGK in B-cells for the high mutation rates and special mutation hotspots at serine codons. Findings of the distinct Ig repertoire in myeloblasts may facilitate the discovery of a new molecular marker for disease monitoring and target therapy.

## 1. Introduction

Acute myeloid leukemia (AML) is a group of genetically heterogeneous diseases characterized by abnormal overproduction of immature myeloid cells, resulting in hematopoiesis impairment and bone marrow failure [1,2]. Despite the advances in combined chemotherapy and hematopoietic stem cell transplantation, the survival rate of AML patients has not been remarkably improved in the past years. It has been reported that molecular genetic aberrations can be detected in approximately 90% of AML patients [3,4]. AML patients can be stratified into favorable, intermediate, or adverse prognostic risk groups based on their cytogenetic and mutation profile. Thus far, about sixty mutations in genes, such as FLT3, NPM1, CEBPA, IDH1, IDH2, KIT, RUNX1, and TP53, have helped further refine risk stratification and precise therapy [5,6]. Therefore, identifying new molecular targets is of great importance for not only guiding risk stratification, but also monitoring minimal/measurable residual disease (MRD) and developing targeted therapy.

Monoclonal immunoglobulin (Ig) gene rearrangement has been used as a diagnostic tool for B-cell lymphomas [7]. Although B-cells and plasma cells have been considered as the only source of Ig, a series of reports from our group and others have demonstrated that Ig could be expressed in many epithelial cancer cells [8], as well as in normal non-hematopoietic cells [9,10,11]. Moreover, other than acting as natural antibodies [12,13], non-B-derived Ig, especially IgG, with unique rearrangement patterns or glycosylation profile, has been shown to play a role in promoting cancer cell survival and proliferation, as well as cancer invasion and metastasis [8,14]. Non-B-derived Ig was frequently expressed in hematopoietic cells, including umbilical cord CD34^+^ stem/progenitor cells (IgM) [15], AML blasts (IgG, IgM, and Igκ) [16,17,18], and mature myeloid cells (IgM, and Igκ) [17,18]. The expression of AML-derived Ig correlated with cell viability, proliferation, and migration [16,17,18]. Furthermore, AML patients with blasts showing high levels of IgG staining signals by flow cytometry had significantly poorer overall survival [19].

Immunoglobulin consists of two identical heavy chains (IgH) and light chains (IgL). There are five classes of IgH, including IgM, IgG, IgA, IgD, and IgE. The two types of IgL are Igκ and Igλ. Each of the IgH and IgL chains contains a variable region and a constant region. The variable (V) region of the IgH gene is formed by combining IGHV, IGHD, and IGHJ genes, whereas the V region of the IgL gene is generated by combining an IGKV with IGKJ gene or an IGLV with IGLJ gene. The constant region (IGHM, IGHG, IGHA, IGHD, and IGHE) determines the class of IgH. The diversity of Ig results from the V(D)J recombination and random insertions and deletions of non-templated (N) nucleotide at the junctions. Additional diversity is generated by somatic hypermutation (SHM), which is traditionally believed to occur after activation through exposure to an antigen [20]. The mutation is mostly single-nucleotide substitutions introduced by activation-induced cytidine deaminase (AID).

In this study, IGHG and IGK expression were quantified in sorted myeloblasts from 59 AML patients by quantitative real-time polymerase chain reaction (qPCR). We assessed mRNA expression levels and correlated them with patients’ clinical features and prognosis. The higher expression level of AML-derived IgG transcripts correlated with shorter disease-free survival (DFS). Since the function of the Ig is highly correlated with its V region characteristics, we performed a comprehensive sequencing analysis of AML-derived Ig repertoire in 16 patients. All five classes of Ig heavy chain and two types of Ig light chain were amplified for each sample by combined multiplex PCR and next-generation sequencing (NGS). Our results demonstrated the expression of Ig with individually biased repertoire and specific SHM hotspots in myeloblasts, opening a potential venue for AML monitoring and precision therapy.

## 2. Materials and Methods

### 2.1. Patient Samples

Peripheral blood or bone marrow specimens were obtained from AML patients seen at the University of Texas MD Anderson Cancer Center, with written informed consent. The study was conducted according to an Institutional Review Board-approved protocol of the MD Anderson Cancer Center. The clinical data were obtained by review of medical records.

### 2.2. Fluorescence-Activated Cell Sorting (FACS)

**Figure 1:**
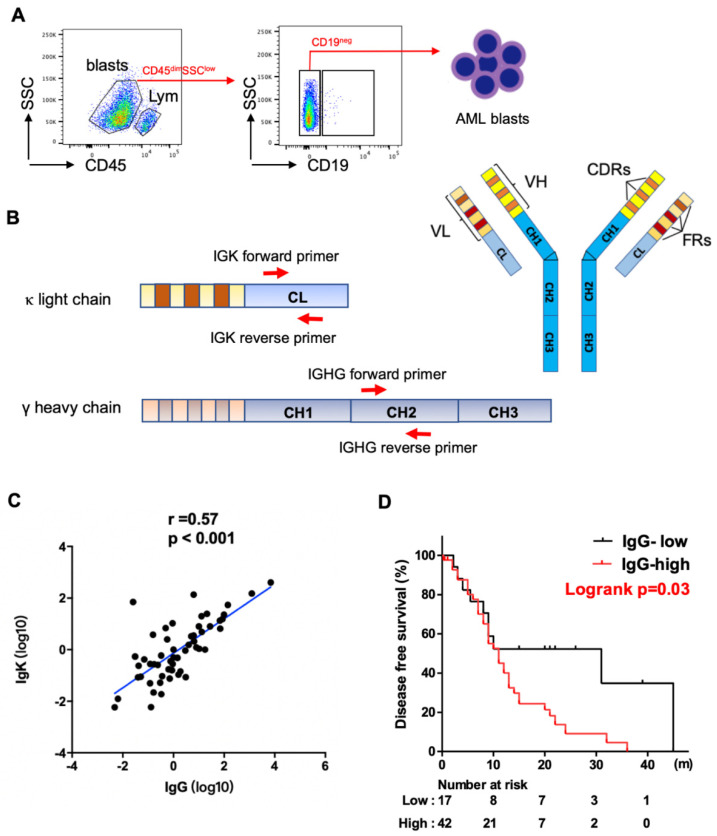
Expression of IGHG and IGKC in AML blasts. (**A**) Schematic map for flow cytometry sorting strategy of AML samples. Blasts: CD45^dim^SSC^low^, lym: CD45^high^SSC^low^ lymphocytes. CD19 was further used to exclude B cell contamination from AML blasts. (**B**) Schematic map of Ig structure and primers complementary to IgG and Igκ constant region used for qPCR analysis. IGHV: variable region of Ig heavy chain, VL: variable region of Ig light chain, CH: constant region of Ig heavy chain, CL: constant region of Ig light chain, FR: framework regions, CDR: complementary determining regions. (**C**) Spearman correlation analysis shows a strong correlation between levels of IGHG and IGKC expression. (**D**) Kaplan–Meier analysis shows higher levels of IGHG expression correlate with shorter disease-free survival in AML patients.

For sorting myeloblasts from AML patients, mononuclear cells were isolated from peripheral blood or bone marrow by Ficoll–Hypaque (GE Healthcare Bioscience, Chicago, IL, USA, Catalog Number. 17-5442-02) density gradient centrifugation. The mononuclear cells were stained with two monoclonal antibodies: anti-CD45-APC-Vio770 (Miltenyi Biotec, Auburn, CA, USA, Catalog Number. 130-096-609), and anti-CD19-APC (Miltenyi Biotec, Catalog Number. 130-091-248) at 4 °C for 30 min. After washing with PBS (supplemented with 2% FBS) three times, the cells were sorted using a FACScan instrument (BD Aria II, Franklin Lakes, NJ, USA). We gated on myeloblasts (CD45-dim, SSC-low), excluding lymphocytes (CD45-high, SSC-low). We then used CD19 to further exclude B-cell contamination from the blasts (Figure 1A).

### 2.3. Quantitative Real-Time Polymerase Chain Reaction (PCR)

Total RNA was isolated from sorted cells using TRIzol reagent (Thermo Fisher, Waltham, MA, USA). First-strand cDNA was synthesized from total RNA using oligo dT(15) primers and Moloney murine leukemia virus reverse transcriptase (MMLV; Invitrogen, Waltham, MA, USA). Quantitative real-time PCR was performed using SYBR Green PCR Master Mix (Thermo Fisher) on ABI 7500 System (Thermo Fisher). The expression of the target gene was normalized to that of glyceraldehyde 3-phosphate dehydrogenase (GAPDH). Fold change was calculated by the 2^−ΔΔCt^ method where ΔCt = Ct(Target) − Ct(Reference). The primers for IGHG constant regions were 5′-ACTACAAGACCACGCCTCC-3′ and 5′-CGTCGCACTCATTTACCC-3′. The primers for IGK constant regions were 5′-CTGTCTTCATCTTCCCGCCA-3′ and 5′-CTTGCTGTCCTGCTCTGTGA-3′.

### 2.4. Next-Generation Sequencing (NGS)

**Figure 2:**
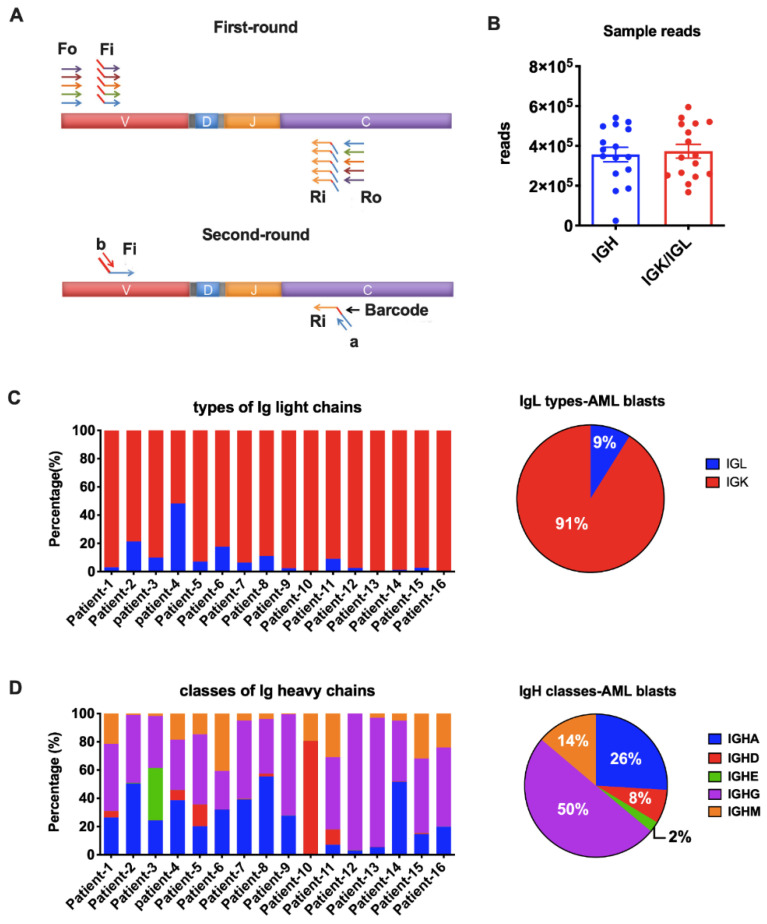
Amplification of Ig heavy chains (IgH) and light chains (IgL). (**A**) Arm-PCR technology was used to amplify the immune repertoire. During the first round PCR, forward primers Fo (forward-out) and Fi (forward-in) were used to target V genes and reverse primers Ro (reverse-out) and Ri (reverse-in) were used to target C genes. The Fi and Ri primers included sequencing adaptors b and a, respectively. There were also barcodes in-between primer a and C gene-specific primers. The second-round PCR was carried out with communal primers b and a. The five classes of IgH were amplified with the same V primer sets but different C primers in one tube for each sample. The two types of IgL were amplified with their own V and C primers in one tube for each sample. (**B**) Total reads for IgH and IgL libraries in NGS (Illumina MiSeq, 2 × 250 bp). (**C**) Proportions of the two types of Ig light chains in each AML sample (**left**) or on average (**right**). (**D**) Proportions of the five IgH classes in each AML sample (**left**) or on average (**right**).

From a total of 59 patients, we performed immune repertoire sequencing analysis on 16 patients with relatively higher levels of Ig expression based on qPCR results and adequate mRNA quality and quantity. For each sample, 100ng RNA was pooled to generate the NGS library. Primer sets for IgH (iRepertoire, Inc., Huntsville, AL, USA, Catalog Number. HBHI-M) and IgL (iRepertoire Inc., Catalog Number. HBKLI-M) were used to perform two rounds of PCR under the reaction conditions specified by iRepertoire^®^. During the first round, reverse transcription was completed, and nested gene-specific primers complementary to V and C genes were used to introduce barcodes and sequencing primers into PCR products. The second-round PCR was carried out using communal (sequencing) primers for exponential amplification. Therefore, the entire repertoire was amplified evenly and semi-quantitatively, without introducing additional amplification bias (Figure 2A). The subsequent quantification, pooling, quality control, and sequencing using the 2 × 250 bp Illumina MiSeq platform were performed by iRepertoire^®^.

### 2.5. Data Analysis

The iRepertoire^®^ company provided the basic data analysis, including barcode de-multiplexing and filtering, V, D, J, C mapping, complementary determining regions (CDR) identification, CDR3 length distribution, and CDR3 algebra (compare repertoires and identify shared CDR3s). The analyzed data in CSV format were then given to us for further analysis using Python and MATLAB programs. Each IgH class or IgL type frequency was calculated based on its reads divided by total reads achieved in the sample. For SHM analysis, filtered DNA sequences were converted to FASTA format and then uploaded to the IMGT/High V-Quest web-based analysis tool [21]. The analyzed IMGT mutation files were used to calculate mutation rates and locate SHM hotspots. Data rendering and mapping were completed by GraphPad Prism 6 software. We used iR-Seq results of IgH provided by iRepertoire^®^ as the control in data analysis, which was amplified from a mixture of total RNA from the peripheral blood mononucleated cells (PBMCs) of 40 healthy people, with the same primer sets and process flow. Similarly, the normal IGK data was amplified from a mixture of total RNA from PBMCs of 426 healthy people.

### 2.6. Statistical Analysis

Statistical analyses were performed using GraphPad Prism 6 software and SPSS16. ROC curves were created by plotting sensitivity (Se(c)) and 1-specificity (1-Sp(c)) for all possible threshold values (c) of a marker. The Youden index, where the threshold value for which Se(c) + Sp(c) − 1 is maximized, was then used to provide a criterion for choosing the optimal cutoff value (c*). The cutoff values for immunoglobulin expression were determined by the Youden Index derived from ROC curves. Spearman correlation analysis was used to evaluate correlations among groups assigned, based on two different immunoglobulins (IgG, Igκ). DFS was calculated from the date of initial diagnosis to the date of relapse, death, or last follow-up. DFS was analyzed using the Kaplan–Meier method and compared using the log-rank test. A *p* value of <0.05 was considered statistically significant. Clinicopathologic parameters and gene mutation status between the two groups (high versus low expression) for each immunoglobulin were compared by Pearson’s chi-square test or Fisher exact test.

## 3. Results

### 3.1. Correlation between Levels of Ig Expression and Clinicopathologic Features

We have detected Igs on the cell surfaces of myeloblasts by multicolor flow cytometry [19]. High levels of Ig staining signals were associated with poor overall survival. Here, we further evaluated the expression of Ig transcripts in myeloblasts from 59 AML patients. The patients included 27 men and 32 women, with a median age of 64 years (range, 24–89) at the initial diagnosis. The laboratory data in 59 patients are shown in Table S1. Dysplasia in at least one of the lineages was noted in 38 (64%) cases. All patients were treated with multi-agent chemotherapy. Eleven (19%) patients also received allogeneic stem cell transplantation.

We obtained myeloblasts (CD45-dim, SSC-low) from the 59 patients, excluding lymphocytes (CD45-high, SSC-low), especially CD19^+^ B-cells by fluorescence-activated cell sorting (FACS) (Figure 1A). To assess the levels of expression of IgG and Igκ, we designed specific intron-spanning primers complementary to the constant regions of IGHG and IGK (Figure 1B). We found that both IgG and Igκ were frequently expressed at the transcriptional level in myeloblasts, with a strong correlation between their expressions using Spearman analysis (*p* < 0.001, Figure 1C).

We further investigated the clinical significance of IgG expression. Using the Youden index and ROC curve analysis, the cutoff value for IgG was determined as 0.16, dividing the patients into IgG high- and low-expression groups. Patients with high IgG expression had a higher proportion of older patients (≥60, *p* = 0.047). No statistically significant differences were noted between the two groups in regards to other clinicopathologic features assessed, including gender, white blood cell (WBC), hemoglobulin level, platelet count, absolute monocyte count, serum level of lactate dehydrogenase (LDH), dysplasia, and WHO classification (Table S2). Using Kaplan–Meier analysis and the log-rank test, AML patients with blasts showing high levels of IgG expression had significantly shorter DFS than those with low levels of IgG expression (Figure 1D). The median DFS was 11 months and 31 months for patients with high versus low expression, respectively (*p* = 0.03). NGS mutation analysis was performed on all patients using a panel of 81 genes that are commonly mutated in hematopoietic neoplasms. The analysis detected at least one gene mutation in 57 (97%) cases. High levels of IgG expression were associated with KIT mutations (*p* = 0.02) and NPM1 mutations (*p* = 0.03) (Table S3). All ten *NPM1* mutated cases were in the IgG-high expression group, and among these ten cases, six were accompanied with FLT3-ITD mutation, one with FLT3-TKD mutation, one with both, and two were FLT3 unmutated.

### 3.2. Detecting AML-Derived Ig Repertoire by NGS

The huge diversity is a unique feature of B cell-derived Igs, which function as antibodies against various antigens. Our previous studies have shown that cancer-derived Ig V genes have important tumor biological activities. Since the function of Ig is mainly determined by the characteristics of V region, we explored the signature of AML-derived Ig sequences. We selected myeloblasts from 16 AML patients (10 men and 6 women) and performed a comprehensive sequencing analysis of both IgH and IgL repertoire by combined multiplex PCR (Figure 2A) and NGS (Illumina MiSeq, 2 × 250 bp). The median age at the initial diagnosis was 64 years (range, 31–85). Most patients were diagnosed as acute myelomonocytic leukemia (FAB-M4, 7 cases) or acute monoblastic/monocytic leukemia (FAB-M5, 4 cases), one patient had AML without maturation (FAB-M1), and the other four patients presented to us as relapsed AML. None of the patients with abnormal karyotype had aberrations involving chromosomal regions close to where IGH (14q32), IGK (2p11), or IGL (22q11) genes are located (Table S4).

We amplified all five classes of heavy chain (IGHA, IGHD, IGHE, IGHG, and IGHM) and two types of light chain (IGK and IGL) in the blasts from the 16 AML patients. An average of 350,000 Ig heavy chain sequences and 370,000 Ig light chain sequences were obtained for each sample by NGS (Figure 2B). The IGK and IGL genes are located on chromosomes 2 and 22, respectively. It is known that rearrangement at the IGK locus is activated once the IGH genes have rearranged. In B cells, gene arrangement proceeds at the IGL locus only when functional rearrangement is not achieved on either IGK allele [22,23]. However, it is still unclear if Ig gene rearrangements are subject to the same mechanism in non-B cells. As reported in normal B cells, the κ to λ ratio is 60:40 [24]. In fact, a κ/λ ratio of >3:1 or <0.3:1 has been used as a criterion in the diagnosis of B cell lymphomas [25]. In this study, we amplified IGK and IGL in the same tube simultaneously. We found that IGK was expressed much more frequently than IGL (>3:1) in 15/16 AML blasts (Figure 2C), indicating that, similar to B cell-derived Ig, IGK rearrangements occurred prior to IgL. The broken balance between κ to λ ratio reflected the abnormal proliferation of Igκ-expressing myeloblasts in AML patients. IGL had similar reads with IGK only in Patient-4. We hypothesize that this may result from clonal expansion of both κ and λ-expression blasts in this patient. All five IgH classes were successfully detected in AML blasts. In general, IGHG was used most frequently, followed by IGHA and IGHM (Figure 2D). IGHD covered 80% IgH reads in patient-10 and 5~10% in another four patients, but had very low expression in other patients (Figure 2D). IGHE was hardly detected except in patient-2 (Figure 2D).

### 3.3. Biased Ig Rearrangements in AML Blasts

**Figure 3:**
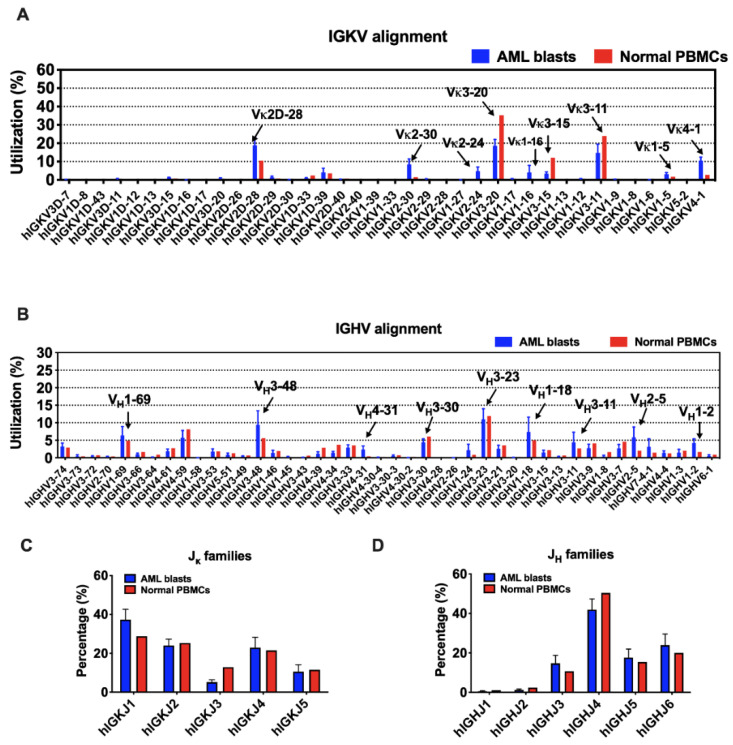
The usage of V and J segments in AML blasts and normal PBMCs. (**A**) Usages of human IGKV gene segments selected by AML blasts and normal PBMCs. The order of IGKV gene segments on the X-axis is according to that on the chromosome. (**B**) Usages of human IGHV gene segments selected by AML blasts and normal PBMCs. The order of IGHV gene segments on the X-axis is according to that on the chromosome. (**C**) Usages of human IGKJ families by AML blasts and normal PBMCs. (**D**) Usages of human IGHJ families by AML blasts and normal PBMCs. Bars indicate mean ± SEM.

Although there are 38 IGKV segments in the human genome, limited segments were used by myeloblasts, as well as normal B cells (Figure 3A). IGKV3-20 and IGKV3-11 were preferred in both AML blasts and normal PBMCs. In addition, IGKV2D-28, IGKV4-1, and IGKV2-30 were more frequently used by myeloblasts, while IGKV3-15 was more preferred by normal PBMCs. In general, the ranking of IGKJ segments used by myeloblasts was IGKJ1, IGKJ2, IGKJ4, IGKJ5, and IGKJ3 (Figure 3C). IGKJ1 was more frequently used and IGKJ3 was less frequently used in myeloblasts compared to normal PBMCs.

In contrast, the usage of IGHV gene segments was more variable. IGHV3-23 was a ‘public’ usage widely expressed not only in normal PBMCs (mean level, 11.9%), but also in myeloblasts (13 in 16 cases; mean level, 11.0%). However, many other V_H_ segments were used by myeloblasts at levels different from that in normal PBMCs. For example, 14 of 16 AML cases used IGHV3-48 at an average usage of 9.4%, much higher than that in normal PBMCs (5.6%). Overall, IGHV1-69, IGHV1-18, IGHV3-11, IGHV2-5, IGHV1-2, and IGHV4-31 were used more frequently in myeloblasts than normal PBMCs (Figure 3B). Similar to that in normal PBMCs, IGHJ4 was used the most frequently by AML-derived Igs, followed by IGHJ6, IGHJ5, and IGHJ3, while IGHJ1 and IGHJ2 were barely used (Figure 3D).

Theoretically, the 43 IGK genes (38 IGKV and 5 IGKJ) and 73 functional IGH genes (44 IGHV, 23 IGHD, and 6 IGHJ) can generate 190 (38 × 5) possible IGK and 6072 (44 × 23 × 6) possible IGH rearrangement patterns, respectively. However, Ig rearrangement patterns used by myeloblasts were biased individually, which means that the blasts from each AML patient had their dominant V(D)J patterns. Interestingly, we found that different patients frequently shared three Vκ-Jκ patterns: IGKV3-20*01/IGKJ1*01 (among top 3 in 6/16, mean utilization: 12.9%), IGKV2D-28*01/IGKJ1*01 (among top 3 in 5/16, mean utilization: 23.9%), and IGKV4-1*01/IGKJ1*01 (among top 3 in 5/16, mean utilization: 12.3%) (Table 1). The preferential expression of certain IGKV segments in myeloid cells, such as IGKV2D-28, IGKV4-1, and IGKV2-30, may indicate a risk of occurrence or recurrence of AML. Considering the low usage of Igλ in myeloblasts, we chose four samples whose IGL reads were over 40,000 for the rearrangement analysis. Similarly, the Vλ-Jλ patterns were biased in these samples. In general, IGLV6-57/IGLJ3, IGLV1-51/IGLJ3, and IGLV1-44/IGLJ3 were frequently used in more than one sample (Figure S1). IGLV2-14 and IGLV3-25 segments were also selected frequently in different samples, but with different IGLJs (Figure S1).

The V_H_DJ_H_ patterns in myeloblasts also lacked diversity, with the top 1 used V_H_DJ_H_ having usage of over 30% in some patients (patients 4, 10, 11, 12, 13, and 14). However, we failed to identify any overlapping V_H_DJ_H_ rearrangements that were frequently used by different individuals (Table 2). That is to say, AML-derived Ig presented its unique preference of V_H_DJ_H_ patterns individually, which may serve as a marker for the detection of residual or recurrent disease.

### 3.4. Recurring CDR3s Revealed Clonal Expansion in AML

**Figure 4:**
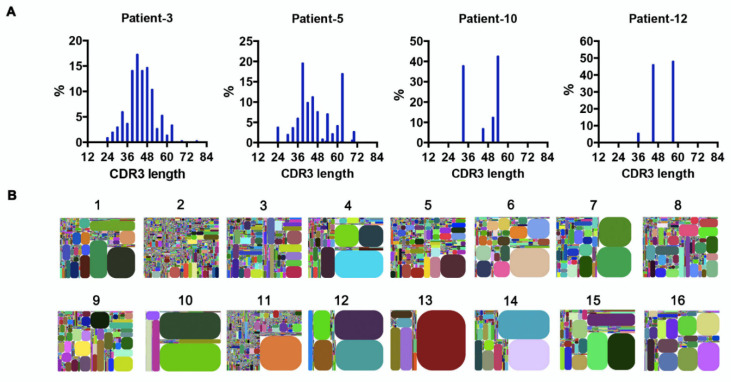
Clonal expansion in AML blasts. (**A**) The distribution of IgH–CDR3 nucleotide length in AML blasts from four representative samples. (**B**) IGHV–IGHJ–CDR3 map of AML blasts (1–16). Each rectangle represents a unique IGHV–IGHJ–CDR3 nucleotide sequence, and the size denotes its relative frequency. Colors for each rectangle are chosen randomly and, thus, do not match between plots.

The random nucleotide addition/deletion process at the joining ends during V(D)J rearrangement contributes to Ig diversity. The CDR3 of the Ig heavy chain, which falls at the V-D-J joining ends, is the most polymorphic region of Ig. Therefore, Ig diversity correlates with IgH-CDR3 variability [26]. Under physiological conditions, the distribution of CDR3 nucleotide length is Gaussian-like [27]. However, we found that in AML-derived IgH, the CDR3 length showed a ‘perturbed’ (e.g., patients 3 and 5) or ‘skewed’ (e.g., patients 10 and 12) distribution (Figure 4A), indicating potential ‘oligoclonal’ or ‘monoclonal’ Ig sequences [27]. In addition, we generated CDR3 tree-maps for samples from each patient (Figure 4B), in which each spot represented a unique entry, IGHV–IGHJ–CDR3, and the size of the spot denoted relative frequency. The tree-maps demonstrated that AML-derived Ig exhibited a high frequency of recurring JGHV–JGHJ–CDR3 usage (Figure 4B), reflecting the clonal expansion of myeloblasts in AML patients. Previous studies in patients with chronic lymphocytic leukemia/small lymphocytic lymphoma (CLL/SLL) revealed conserved CDR3 sequences in different patients, which were expected to serve as therapeutic targets [28]. In this study, despite the high frequency of recurring CDR3 sequences in individuals, no shared CDR3 sequences of IgH were found in the AML patients.

### 3.5. Frequent Somatic Hypermutations Occurred in AML-Derived Igs

**Figure 5:**
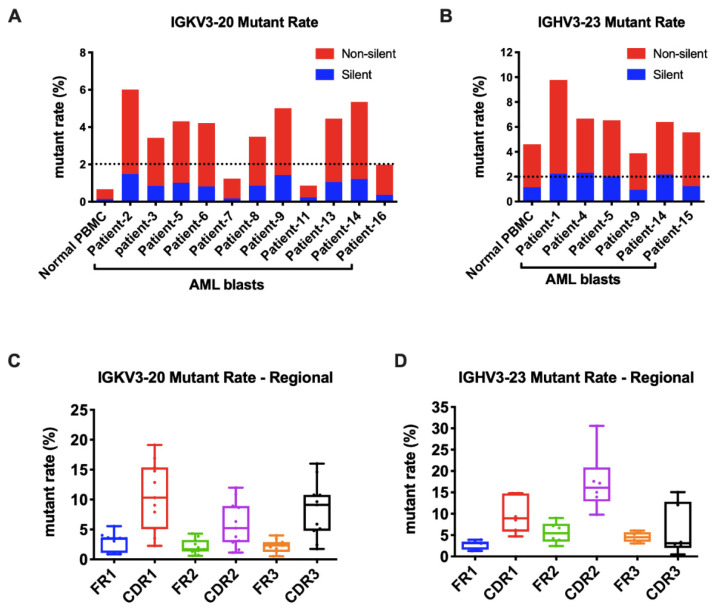
AML-derived Igs undergo somatic hypermutations. (**A**) The mutation rates of Vκ3-20 in normal PBMCs and AML blasts. A cut-off rate of 2% is shown by the dotted line. (**B**) The mutation rates of IGHV3-23 in normal PBMCs and AML blasts. A cut-off rate of 2% is shown by the dotted line. (**C**) The mutation rates of framework regions (FRs) and complementary determining regions (CDRs) in AML-derived IGKV3-20. (**D**) The mutation rates of FRs and CDRs in AML-derived IGHV3-23.

As known, the Ig gene undergoes SHM, affecting its variable region genes upon antigen stimulation. In B cells, SHM increases the affinity of the antibody. We further explored if SHM also occurred in AML-derived Igs. The mutation status is designated as unmutated if there are <2% mutations and as mutated if there are ≥2% mutations compared to the germline sequences. For Igκ, we analyzed the widely shared IGKV3-20 and IGKV2D-28 as models. Surprisingly, in normal PBMCs, they were both conservative without SHM. However, in AML blasts, IGKV3-20 sequences were mutated in 9/11(82%) samples (Figure 5A). Similarly, IGKV2D-28 displayed SHM in 9/10 (90%) samples (Figure S2A). By analyzing the most commonly used IGHV3-23 and IGHV3-48, we found that SHM frequently occurred in normal PBMCs and myeloblasts (6/6 for IGHV3-23 and 3/4 for IGHV3-48) (Figure 5B and Figure S2B). More than half of the mutations of AML-Ig were nonsynonymous. The mutations of AML-derived Igs occurred at higher frequencies in CDR regions than in framework (FR) regions (Figure 5C,D and Figure S2C,D), similar to those in B cells, indicating a similar underlying regulation mechanism.

### 3.6. AML-Derived IGKV3-20 Displayed Specific Mutation Hotspots at the Serine Codons

**Figure 6:**
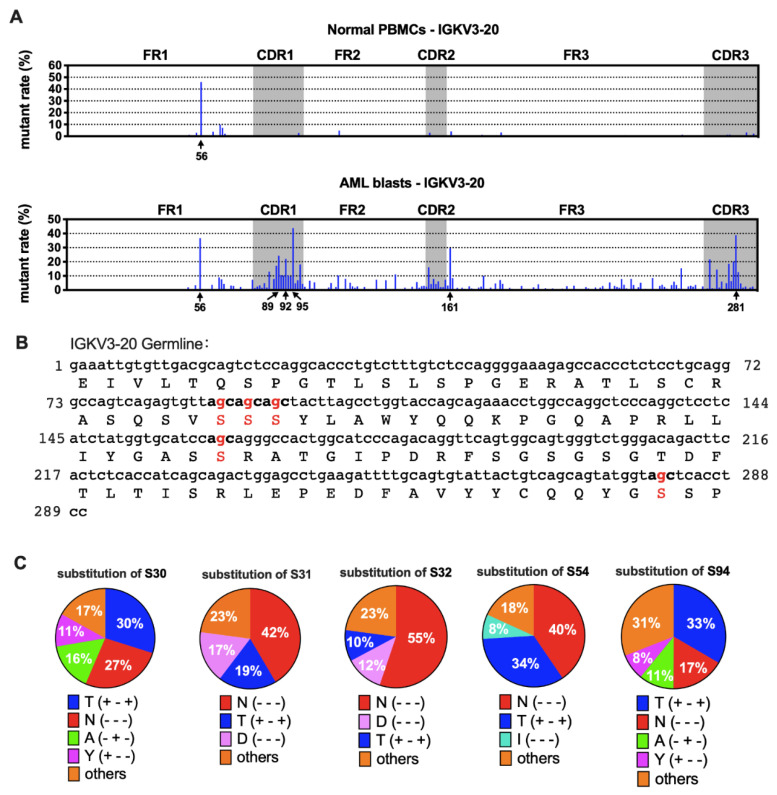
Mutation hotspots in IGKV3-20. (**A**) The mutation rates of Vκ3-20 nucleotides in normal PBMCs (**upper**) and AML blasts (**lower**). (**B**) Germline IGKV3-20 nucleotide and amino acid sequences. The frequently mutated nucleotides and amino acids are highlighted in red. (**C**) Substitutions of serine in the five mutation hotspots. The percentages shown are averages of AML blasts. The amino acid changes are indicated with “+” (same) or “-“ (different) in the parentheses in the following order: hydropathy, volume, and chemical characteristics [29]. For example, T (+ - +) indicates that the two amino acids, S and T, belong to the same hydropathy class and have the same chemical characteristics, but the volume is different.

Furthermore, we compared the sequences of AML-derived Ig with the germline sequences from IMGT [21]. We found that the mutation spots for the same V_H_ usage (e.g., IGHV3-23) varied among patients (Figure S3), indicating that SHM spots of IgH were selected randomly in each case. Surprisingly, for IGKV3-20, other than a common hotspot (nt-56), five special hotspots (nt-89, -92, -95, -161, and -281) were observed that were shared by AML blasts, each having a >20% mutation rate on average (Figure 6A). In AML-derived IGKV2D-28, there were five mutation hotspots (nt-90, -95, -150, -286 and -297) compared to normal PBMCs. The average mutation rates were between 15% and 30% (Figure S4A), but they varied among different samples (data not shown).

There were three kinds of gene mutations defined by their different influence on amino acid (AA) sequences: silence mutation (AA not changed), missense mutation (AA changed), and nonsense mutation (stop codon introduced). Most mutations in Vκ were non-silent (Figure 5A and Figure S2A). We further explored the impact of nucleotide (nt) replacement on the AA sequences in myeloblasts. Surprisingly, we found that all five most frequently mutated points in IGKV3-20 were at the second site of the serine (S) codon (Figure 6B). The main substitutions were threonine (T) and asparagine (N) (Figure 6C), indicating that the AA modification changes may affect functions of AML-Igκ. In contrast, mutation hotspots in IGKV2D-28 did not affect identical AA residues (Figure S4B).

## 4. Discussion

We recently reported that detection of a high level of IgG on AML blasts by flow cytometry analysis, coupled with Igκ or Igλ, is associated with poor overall survival [19]. Considering that AML-derived Ig could not only be on the cell membrane, but may also be located in the cytoplasm or secreted into the circulation, we further assessed AML-derived Ig expression in sorted myeloblasts by qPCR. Similarly, we found that AML patients with a higher level of AML-derived IgG expression at mRNA levels had significantly shorter DFS. In addition, high IgG expression was associated with KIT mutations and NPM1 mutations. KIT mutations have been reported to be seen in approximately 5% of AML cases and predict an unfavorable prognosis [2]. NPM1 mutations are commonly seen in approximately 30% of adult AML and have been used as a genetic marker for the assessment of MRD [30]. It is important to eradicate NPM1-mutated clones to achieve remission [31]. NPM1 by itself has been associated with a favorable prognosis in patients with AML; however, when present with FLT3-ITD, patients have inferior clinical outcomes [6]. In our study, whereas all ten NPM1-mutated cases were in the IgG-high group, seven cases also harbored FLT3-ITD mutation, indicating that AML derived-IgG might be an unfavorable prognostic factor.

Only IGHG, IGHM, and IGK transcripts were assessed in myeloblasts in previous studies [16,17,18]. This study is the first to demonstrate that all the five classes of Ig heavy chains and two types of Ig light chains could be expressed in non-B cells. IGHG has been reported in various non-B neoplastic cells and has been shown to promote malignant behaviors in cancer cells [8]. Similarly, we detected a high frequency of IGHG expression in myeloblasts. Unexpectedly, IGHA was also expressed at high levels in myeloblasts, which has only been detected in cancer cells by a few groups [32,33,34]. There have been no reports on IGHD and IGHE expression in cancer cells; our results showed that both IGHD and IGHE were expressed, though rarely, in myeloblasts.

We have previously used Sanger sequencing to assess AML-derived Ig rearrangements and found clues for their bias and conservation [16,17,18]. In this study, we performed a comprehensive survey of Ig repertoire in AML by NGS. IGKV usage has been shown to be biased in normal B cells [35,36], with dominant Vκ segments including IGKV3-20, IGKV3-11, and IGKV3-15. Restricted light chain diversity has also been reported in neonatal B-cells from cord blood [37,38]. In keeping with these findings, AML-derived Igκ also showed biased rearrangements. Different patients frequently shared three rearrangement patterns of Igκ: IGKV3-20*01/IGKJ1*01, IGKV2D-28*01/IGKJ1*01, and IGKV4-1*01/IGKJ1*01. On the contrary, IgH rearrangements are always in great diversity in normal B cells to recognize various antigens [39]. Surprisingly, AML-derived IgH showed much less diversity, and the dominant rearrangement patterns were very individualized. We could not find a common dominant pattern among different AML samples. This is different from our previous studies of epithelial cancer cell-derived IgH, in which we identified highly conserved IgH sequences [40], showing IGHV3-15/IGHD3-10/IGHJ4, IGHV6-1/IGHD6-3/IGHJ4, and IGHV4-30/IGHD3-22/IGHJ4 mainly used by IGHM, and IGHV5-51/IGHD3-9/IGHJ4 frequently used by IGHG. Nevertheless, we found some preferentially used IGHV fragments in AML, such as IGHV3-23, IGHV3-48, and IGHV1-69, which partially resembled those in CLL/SLL (IGHV1-69, IGHV3-07, IGHV3-23, and IGHV4-34) [41,42,43,44,45,46,47,48], suggesting that Ig tends to have lineage-specific biased usages.

It is known that SHM in B-cells is mediated by AID [49,50]. Whereas AIDs tend to mediate Ig mutations, they can also mediate mutation of other genes, leading to B-cell lymphomas or other malignancies [51]. We do not know if AIDs also participate in SHM of AML-derived Ig or if the instability of the tumor genome induces the high mutation rate of AML-Ig. However, our results provide a series of clues for further research on the regulation mechanisms of non-classical Ig expression.

In this study, five special mutation hotspots were identified in AML-derived IGKV3-20 (nt-89, -92, -95, -161, and -281). Compared with IGKV2D-28, whose expression and mutation rates varied among different patients, we think that IGKV3-20 may serve as a more suitable target with specific imprints of myeloblasts for tracking AML MRD. More surprisingly, we found that IGKV3-20 mutations preferentially caused replacements of serine (S), which is one of the three AA residues commonly phosphorylated by kinases during cell signaling in eukaryotes. Moreover, it can undergo O-linked glycosylation. Therefore, the replacements of serine may lead to a change in Igκ functions via phosphorylation or O-glycosylation. The main substitutions were Asn (N) and Thr (T). The change to Asn (S30N and S32N), which is very dissimilar to Ser in hydropathy, volume, and chemical characteristics, could bring in classic (N-X-T/S) or non-classic (T/S-X-N) N-glycosylation. It is known that glycosylation is essential for the function of Ig (e.g., N-glycosylation at Asn297) [52,53]. We have previously identified a new N-glycosylation at Asn162 carrying a sialic acid modification in the CH1 domain of cancer-derived IgG heavy chain [54]. The protumorigenic role and immunosuppressive function of cancer-derived IgG highly depend on this sialylation modification [55,56]. Further study is needed to evaluate the impact of the amino acid modification on the functions of AML-Ig.

## 5. Conclusions

In summary, we have found that all the five classes of Ig heavy chains and two types of Ig light chains could be expressed in AML blasts, and that a higher level of AML-derived IgG expression correlated with significantly shorter DFS. AML-derived Igs had biased as well as individualized variable region sequences. Compared to AML-derived IgH, AML-derived IGK was more conserved among different AML samples. Moreover, it differed from classical IGK in B-cells in their hypermutation rate and special mutation hotspots at the serine site. AML-derived Ig, especially Igκ, may play a role as a novel AML-related gene that contributes to leukemogenesis and AML progression. It may also serve as a molecular marker for MRD monitoring, risk stratification, and the development of target therapy.

## Figures and Tables

**Table 1:** The top three Vκ-Jκ patterns used by AML blasts.

PID *	Vκ	Jκ	Utilization (%)	PID *	Vκ	Jκ	Utilization (%)
	IGKV2-30*02	IGKJ5*01	39.4		IGKV2D-28*01	IGKJ1*01	27.3
1	IGKV4-1*01	IGKJ1*01	7.8	9	IGKV2D-28*01	IGKJ3*01	10.5
	IGKV3-15*01	IGKJ2*01	6.8		IGKV3-20*01	IGKJ2*01	7.7
	IGKV3-20*01	IGKJ1*01	14.4		IGKV3-11*01	IGKJ4*01	56.4
2	IGKV3-20*01	IGKJ3*01	6.5	10	IGKV2D-28*01	IGKJ2*01	18.2
	IGKV2D-28*01	IGKJ5*01	6.0		IGKV1-5*03	IGKJ2*01	9.1
	IGKV2-24*01	IGKJ1*01	20.4		IGKV2D-28*01	IGKJ1*01	11.6
3	IGKV3-20*01	IGKJ4*01	10.3	11	IGKV3-20*01	IGKJ1*01	11.4
	IGKV4-1*01	IGKJ2*01	9.8		IGKV4-1*01	IGKJ1*01	8.7
	IGKV3-11*01	IGKJ4*01	55.5		IGKV1-16*02	IGKJ1*01	55.7
4	IGKV1-5*03	IGKJ1*01	3.9	12	IGKV1D-39*01	IGKJ2*01	31.9
	IGKV4-1*01	IGKJ1*01	3.6		IGKV1D-39*01	IGKJ1*01	5.8
	IGKV2-24*01	IGKJ1*01	28.1		IGKV3-20*01	IGKJ4*01	14.5
5	IGKV2D-28*01	IGKJ1*01	6.1	13	IGKV2D-28*01	IGKJ1*01	13.5
	IGKV3-20*01	IGKJ1*01	4.3		IGKV3-11*01	IGKJ5*01	8.5
	IGKV2-30*02	IGKJ4*01	19.7		IGKV2D-28*01	IGKJ1*01	60.9
6	IGKV3-20*01	IGKJ1*01	10.0	14	IGKV3-20*01	IGKJ5*01	16.0
	IGKV3-11*01	IGKJ4*01	9.8		IGKV4-1*01	IGKJ1*01	13.0
	IGKV2D-28*01	IGKJ2*01	24.9		IGKV2D-28*01	IGKJ2*01	33.2
7	IGKV3-20*01	IGKJ2*01	20.4	15	IGKV4-1*01	IGKJ1*01	28.8
	IGKV2D-28*01	IGKJ3*01	8.2		IGKV2-30*01	IGKJ1*01	14.9
	IGKV3-20*01	IGKJ4*01	20.0		IGKV3-11*01	IGKJ1*01	18.1
8	IGKV3-20*01	IGKJ1*01	17.6	16	IGKV3-20*01	IGKJ1*01	16.6
	IGKV3-20*01	IGKJ2*01	14.7		IGKV2-30*02	IGKJ1*01	12.0

* PID: patient ID. The Vκ-Jκ patterns frequently shared among different patients are marked in bold font and colored. Different colors represent different Vκ-Jκ patterns.

**Table 2:** The top three V_H_DJ_H_ patterns used by AML blast.

Patient ID	V_H_	D	J_H_	Utilization (%)
	IGHV3-23*04	IGHD3-3*02	IGHJ6*03	19.3
1	IGHV4-59*01	IGHD2-15*01	IGHJ3*02	14.7
	IGHV1-2*02	IGHD7-27*01	IGHJ3*02	10.0
	IGHV1-18*01	IGHD3-3*02	IGHJ6*02	4.7
2	IGHV3-74*03	IGHD1-7*01	IGHJ4*02	2.4
	IGHV1-18*01	IGHD3-10*01	IGHJ6*03	1.7
	IGHV4-61*08	IGHD6-13*01	IGHJ4*02	4.4
3	IGHV4-4*07	IGHD3-10*02	IGHJ3*02	4.4
	IGHV3-9*01	IGHD5-5*01	IGHJ4*02	4.3
	IGHV2-5*10	IGHD3-9*01	IGHJ5*02	32.0
4	IGHV7-4-1*02	IGHD6-19*01	IGHJ4*02	12.1
	IGHV3-23*04	IGHD3-16*02	IGHJ4*02	11.9
	IGHV3-23*04	IGHD1-7*01	IGHJ4*02	13.2
5	IGHV1-2*02	IGHD1-7*01	IGHJ4*02	2.9
	IGHV1-46*01	IGHD6-13*01	IGHJ4*02	2.7
	IGHV3-48*03	IGHD3-16*02	IGHJ5*02	25.2
6	IGHV1-2*02	IGHD3-10*01	IGHJ4*02	10.2
	IGHV3-73*02	-	IGHJ6*02	5.8
	IGHV4-59*01	IGHD3-16*01	IGHJ5*02	21.0
7	IGHV4-b*01	IGHD5-5*01	IGHJ4*01	19.0
	IGHV3-30*02	IGHD6-13*01	IGHJ6*03	5.7
	IGHV1-46*03	IGHD2-2*03	IGHJ4*02	7.5
8	IGHV4-59*08	IGHD3-3*02	IGHJ3*02	4.8
	IGHV2-5*10	IGHD3-22*01	IGHJ4*02	4.3
	IGHV3-23*04	IGHD6-19*01	IGHJ4*02	8.4
9	IGHV4-31*03	IGHD3-16*02	IGHJ4*02	6.1
	IGHV3-74*02	IGHD3-22*01	IGHJ4*02	5.4
	IGHV3-48*02	IGHD1-26*01	IGHJ3*02	38.6
10	IGHV2-5*10	IGHD5-5*01	IGHJ5*02	37.4
	IGHV3-30*18	IGHD4-17*01	IGHJ3*02	8.4
	IGHV7-4-1*02	IGHD3-22*01	IGHJ4*02	31.9
11	IGHV3-33*01	IGHD1-20*01	IGHJ4*02	1.3
	IGHV3-33*01	IGHD3-22*01	IGHJ3*02	1.2
	IGHV3-48*03	IGHD6-13*01	IGHJ6*02	34.6
12	IGHV3-11*01	IGHD6-6*01	IGHJ5*02	33.8
	IGHV3-11*01	IGHD6-13*01	IGHJ6*02	13.4
	IGHV1-18*01	IGHD6-19*01	IGHJ6*02	65.8
13	IGHV1-24*01	IGHD3-9*01	IGHJ6*02	11.1
	IGHV1-24*01	IGHD3-10*01	IGHJ6*02	9.3
	IGHV3-23*04	IGHD2-2*03	IGHJ6*03	30.9
14	IGHV1-69*13	IGHD6-13*01	IGHJ4*02	18.5
	IGHV1-69*12	IGHD6-13*01	IGHJ4*02	15.5
	IGHV3-23*04	IGHD3-9*01	IGHJ4*02	22.2
15	IGHV1-69*09	IGHD3-9*01	IGHJ3*01	17.2
	IGHV3-7*02	IGHD6-6*01	IGHJ4*02	12.6
	IGHV3-9*01	IGHD6-19*01	IGHJ6*02	11.8
16	IGHV1-3*01	IGHD3-22*01	IGHJ4*02	11.1
	IGHV1-69*13	IGHD3-3*01	IGHJ3*01	8.7

## Data Availability

The data presented in this study are available upon request from the corresponding authors.

## Supplementary Materials

The following are available online at https://www.mdpi.com/article/10.3390/biology11020161/s1, Figure S1: Heat-map of Vλ-Jλ patterns expressed by AML-blasts; Figure S2: Mutation rates of IGKV2D-28 and IGHV3-48; Figure S3: Location of mutations (using IMGT-numbering) and corresponding frequencies of IGHV3-23 in different patients; Figure S4: Mutation hotspots in IGKV2D-28; Table S1: Laboratory data of 59 AML patients; Table S2: Correlation between IgG expression and clinicopathologic features; Table S3: Correlation between IgG expression and molecular genetic features; Table S4: Clinical information of AML patients in the NGS group.
