# Supplementary material for: Next-Generation Sequencing Revealed a Distinct Immunoglobulin Repertoire with Specific Mutation Hotspots in Acute Myeloid Leukemia
Source: Biology (Basel). 2022 Jan 19;11(2):161. doi: 10.3390/biology11020161 (PMC8869405; doi:10.3390/biology11020161)
Supplement: Supplementary file 1 [file biology-11-00161-s001.zip › Supplementary Figures.pdf]

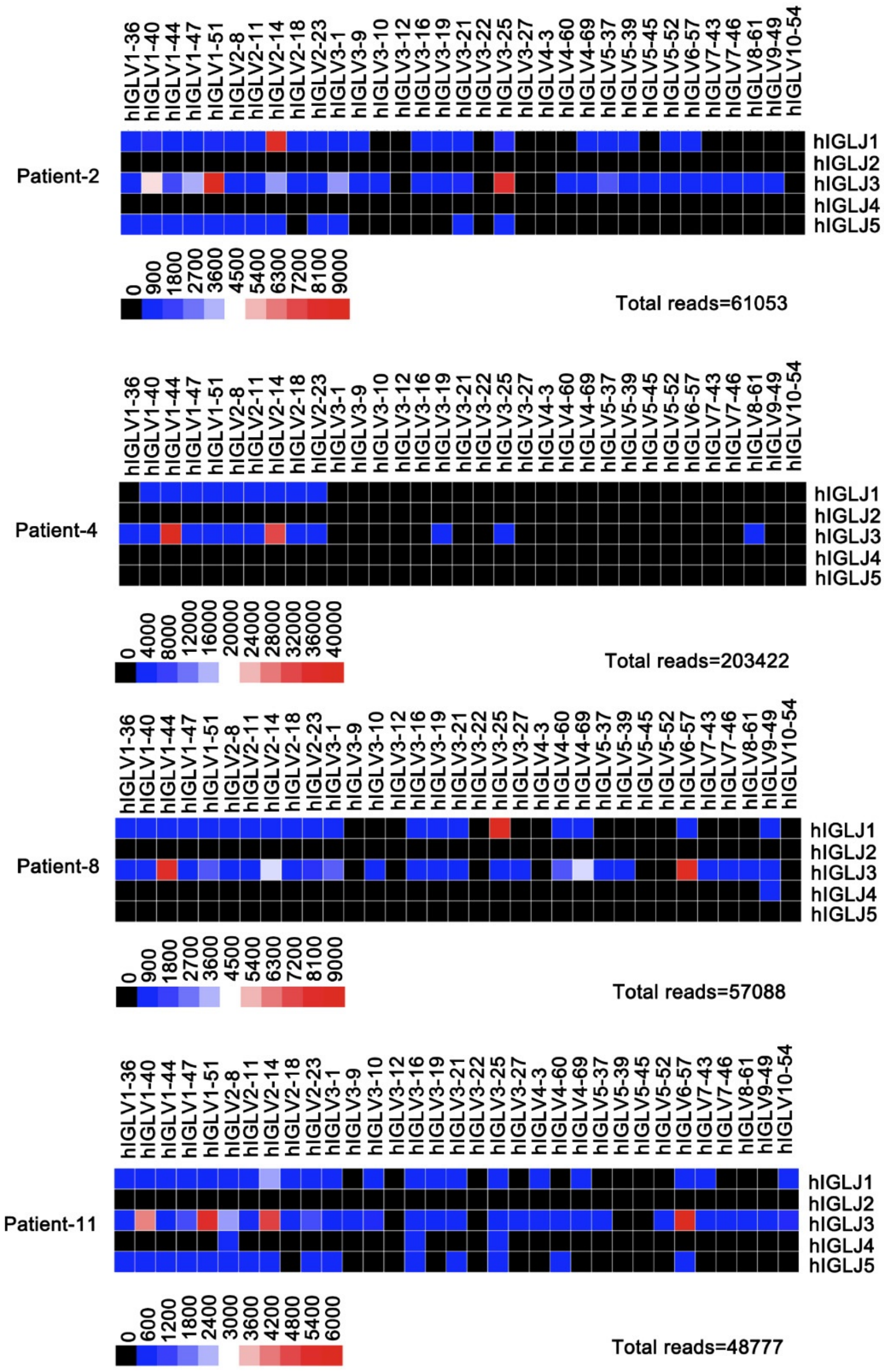

Figure S1. Heat-map of Vλ-Jλ patterns expressed by AML-blasts.

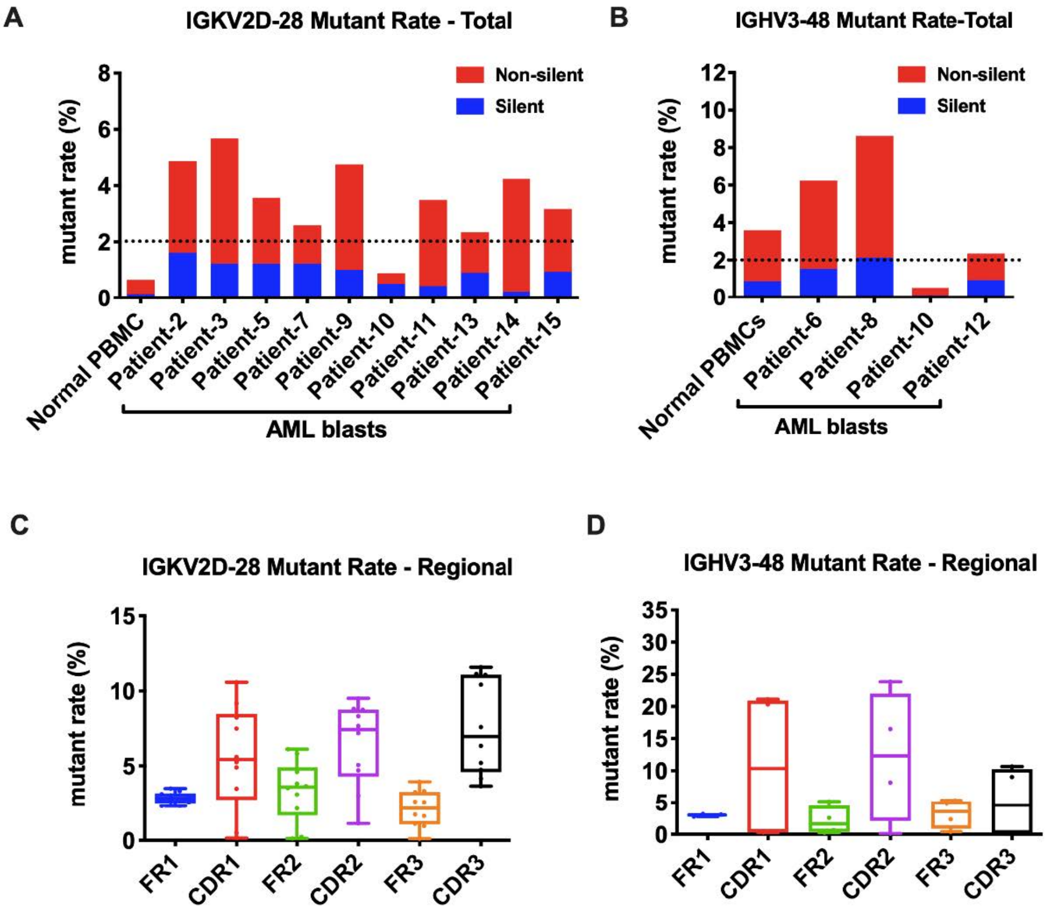

Figure S2. Mutation rates of IGKV2D-28 and IGHV3-48. (A) Mutation rate of IGKV2D-28 in normal PBMCs and AML blasts. A cut-off rate of 2% is shown by the dotted line. (B) Mutation rate of IGHV3-48 in normal PBMCs and AML blasts. A cut-off rate of 2% is shown by the dotted line. (C) Mutation rate of framework regions (FRs) and complementary determining regions (CDRs) in AML-derived IGKV2D-28. (D) Mutation rate of FRs and CDRs in AML-derived IGHV3-48.

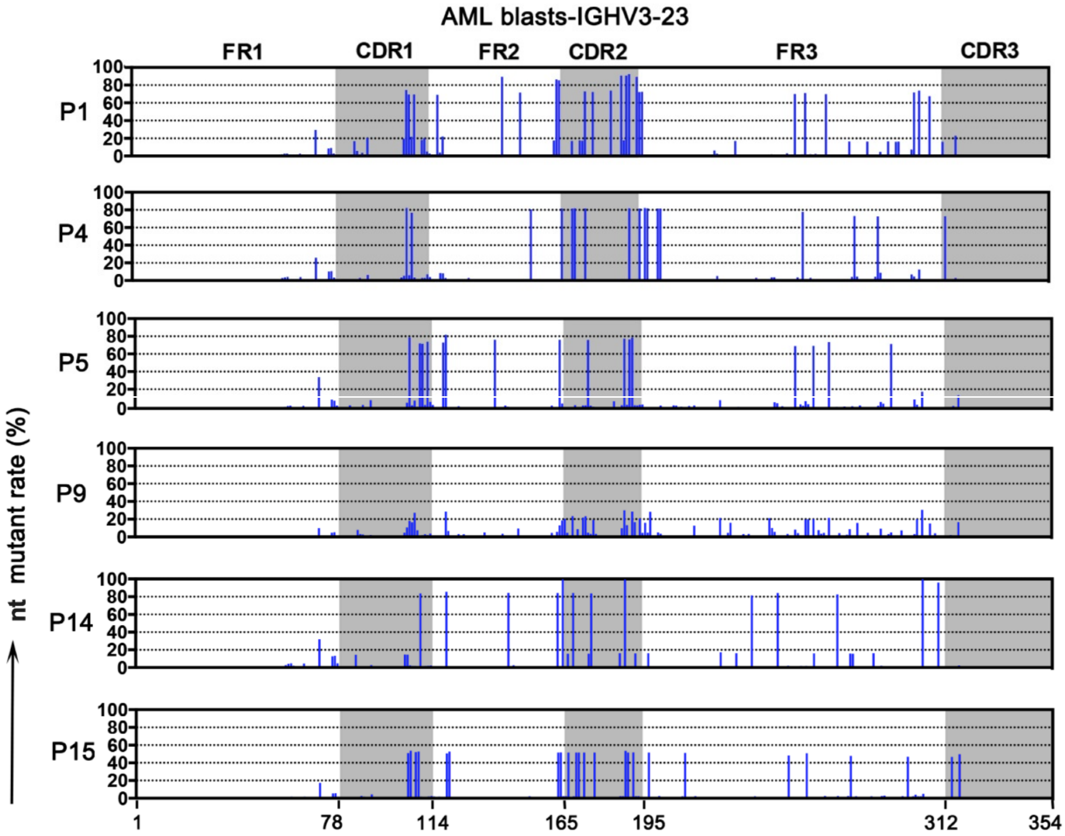

Figure S3. Location of mutations (using IMGT-numbering) and corresponding frequencies of IGHV3-23 in different patients.

A

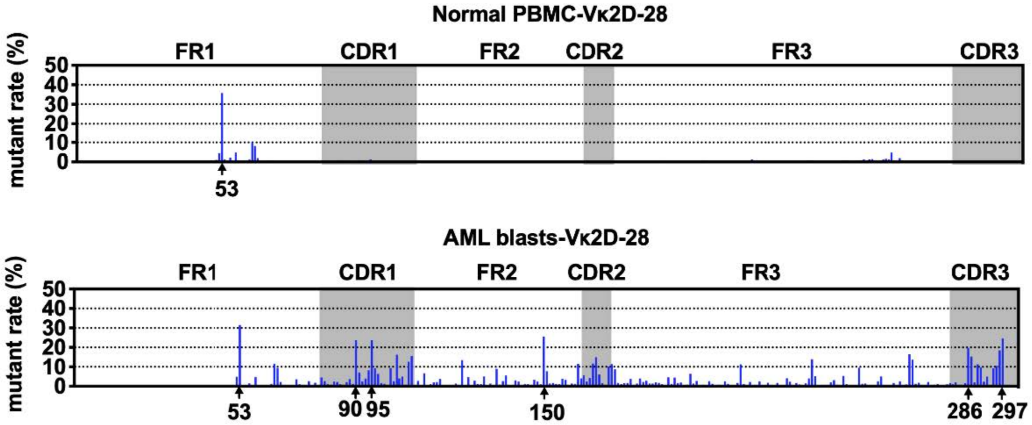

B IGKV2D-28 Germline：

```
  1 gatattgtgatgactcagtctccactctccctgcccgtcacccctggagagccggcctccatctcctgcagg  72
     D  I  V  M  T  Q  S  P  L  S  L  P  V  T  P  G  E  P  A  S  I  S  C  R
 73 tctagtcagagcctcctgcatagtaatggatacaactatttggattggtacctgcagaagccagggcagtct 144
     S  S  Q  S  L  L  H  S  N  G  Y  N  Y  L  D  W  Y  L  Q  K  P  G  Q  S
145 ccacagctcctgatctatttgggttctaatcgggcctccggggtccctgacaggttcagtggcagtggatca 216
     P  Q  L  L  I  Y  L  G  S  N  R  A  S  G  V  P  D  R  F  S  G  S  G  S
217 ggcacagattttacactgaaaatcagcagagtggaggctgaggatgttggggtttattactgcatgcaagct 288
     G  T  D  F  T  L  K  I  S  R  V  E  A  E  D  V  G  V  Y  Y  C  M  Q  A
289 ctacaaactcctcc                                                           305
     L  Q  T  P
```

Figure S4. Mutation hotspots in IGKV2D-28. (A) Mutation rate of Vκ2D-28 nucleotides in normal PBMCs (upper) and AML blasts (lower). (B) Germline IGKV2D-28 and amino acid sequence. The frequently mutated nucleotides and amino acids are highlighted in red.
