# Supplementary material for: Next-Generation Sequencing Revealed a Distinct Immunoglobulin Repertoire with Specific Mutation Hotspots in Acute Myeloid Leukemia
Source: Biology (Basel). 2022 Jan 19;11(2):161. doi: 10.3390/biology11020161 (PMC8869405; doi:10.3390/biology11020161)
Supplement: Supplementary file 1 [file biology-11-00161-s001.zip › Supplementary Tables.pdf]

Table S1. Laboratory data of 59 AML patients.

Laboratory data	Unit	Median	Range	Reference range
WBC	K/uL	16.8	0.4-620.4	4.0-11.0
Hemoglobin	g/dL	8.8	4.6-13.9	14.0-16.0 (men)<br>12.0-14.0 (women)
Platelets	K/uL	58	10-458	140-440
Monocytes	K/uL	0.5	0-73.03	0.08-0.70
LDH	IU/L	798	287-10696	313-618
β2-microglobulin	mg/L	2.7	1.4-8	0.6-2.0

LDH, lactate dehydrogenase; WBC, white blood cells

Table S2. Correlation between IgG expression and clinicopathologic features

	IgG expression		p-value
	**Low**	**High**	
	**N=17**	**N=42**	
**Age** (year)			0.047
<60	10 (59%)	13 (31%)	
≥60	7 (41%)	29 (69%)	
**Gender**			0.26
Male	7 (41%)	20 (48%)	
Female	10 (59%)	22 (52%)	
**WBC** (K/uL)			0.48
Normal	3 (18%)	3 (7%)	
high	10 (59%)	27 (64%)	
Low	4 (24%)	12 (29%)	
**Hemoglobin** (g/dL)			1.00
Normal	0 (0%)	1 (2%)	
Low	17 (100%)	41 (98%)	
**Monocytes** (K/uL)			0.20
Normal	12 (71%)	22 (52%)	
high	5 (29%)	20 (48%)	
**LDH** (IU/L)			0.21
Normal	4 (24%)	15 (36%)	
high	12 (70%)	27 (64%)	
Low	1 (6%)	0 (0%)	
**Dysplasia**			0.08
Yes	8 (47%)	30 (71%)	
No	9 (53%)	12 (29%)	
**WHO classification**			0.18
AML-RGA	3 (18%)	1 (2%)	
AML-MRC	2 (12%)	13 (31%)	
t-MN	4 (23%)	4 (10%)	
AML-M0	1 (6%)	2 (5%)	
AML-M1	3 (18%)	7 (17%)	
AML-M2	0 (0 %)	5 (12%)	
AML-M4/M5	3 (18%)	9 (21%)	
Ph^+ AML	1 (6%)	1 (2%)	

AML, acute myeloid leukemia; AML-M0, AML with minimal differentiation; AML-M1, AML without differentiation; AML-M2, AML with maturation; AML-M4/M5, AML with monocytic differentiation; LDH, lactate dehydrogenase; MRC, myelodysplasia-related changes; RGA, recurrent genetic abnormalities; t-MN, therapy-related myeloid neoplasm; WBC, white blood cells

Table S3. Correlation between IgG expression and molecular genetic features

	IgG expression		p-value
	**Low**	**High**	
	**N=17**	**N=42**	
**Karyotype**			0.51
Diploid	3 (18%)	11 (26%)	
Simple	8 (47%)	22 (53%)	
Complex	6 (35%)	9 (21%)	
**Gene mutations**			0.2
No mutation	0 (0%)	2 (5%)	
Mutations	17 (100%)	40 (95%)	
1-2mutations	13 (77%)	22 (52%)	
≥3mutations	4 (23%)	18 (43%)	
ASXL1			0.26
Wild type	16 (94%)	34(81%)	
Mutated	1 (6%)	8 (19%)	
CEBPA			0.24
Wild type	16 (94%)	41 (98%)	
Mutated	1 (6%)	0 (0%)	
NA	0 (0%)	1 (2%)	
DNMT3A			1.00
Wild type	14 (82%)	33 (78%)	
Mutated	3 (18%)	9 (22%)	
FLT3			0.11
Wild type	13 (75%)	24 (57%)	
ITD	1 (7%)	13 (31%)	
TKD	3 (18%)	3 (16%)	
Both	0 (0%)	2 (3%)	
GATA2			1.00
Wild type	17 (100%)	40 (95%)	
Mutated	0 (0%)	2 (5%)	
IDH1			0.27
Wild type	16 (94%)	35 (83%)	
Mutated	1 (6%)	7 (17%)	
IDH2			0.3
Wild type	14 (82%)	29 (69%)	
Mutated	3 (18%)	13 (31%)	
JAK2			0.57
Wild type	15 (88%)	40 (95%)	
Mutated	2 (12%)	2 (5%)	

Table S3. Correlation between IgG expression and molecular genetic features (continued)

	IgG expression		p-value
	Low	High	
	N=17	N=42	
KIT			0.02
Wild type	15 (88%)	42 (100%)	
Mutated	2 (12%)	0 (0%)	
KRAS			0.11
Wild type	16 (94%)	42 (100%)	
Mutated	1 (6%)	0 (0%)	
MLL			
Wild type	17 (100%)	42 (100%)	
Mutated	0 (0%)	0 (0%)	
MPL			0.29
Wild type	16 (94%)	42 (100%)	
Mutated	1 (6%)	0 (0%)	
NOTCH1			0.50
Wild type	16 (94%)	41 (97%)	
Mutated	1 (6%)	1 (3%)	
NPM1			0.03
Wild type	17 (100%)	32 (76%)	
Mutated	0 (0%)	10 (24%)	
NRAS			0.74
Wild type	14 (82%)	32 (76%)	
Mutated	3 (18%)	10 (24%)	
PTPN11			1.00
Wild type	16 (94%)	38 (91%)	
Mutated	1 (6%)	4 (9%)	
RUNX1			0.74
Wild type	14 (82%)	31 (74%)	
Mutated	3 (18%)	11 (26%)	
TET2			0.71
Wild type	14 (82%)	36 (86%)	
Mutated	3 (18%)	6 (14%)	
TP53			1.00
Wild type	15 (88%)	37 (88%)	
Mutated	2 (12%)	5 (12%)	
WT1			0.62
Wild type	15 (88%)	39 (93%)	
Mutated	2 (12%)	3 (7%)	

Table S4. Clinical information of AML patients in the NGS group

ID	Sex[1]	Age[2]	Clinical Info[3]	FAB	blasts (%)	WBC	Mutation	Karotype	F/U (m)[4]	Out-come[5]
**1**	F	54	nAML	M5b	92	654.1	IDH2, FLT3	46,XX[20]	1	CR
**2**	M	64	nAML[1]	M4	40	80.3	DNMT3A, NPM1, FLT3	46,XY[20]	17	CR
**3**	M	72	pAML	N.A	26	7.4	NRAS, RUNX1	47,XY,+21[17]	10	DOD
**4**	F	55	rAML	M4	72	43.8	DNMT3A, IDH2, NPM1, FLT3	46,XX,t(2;3)(q21;q29)[1]/46,XX,del(3)(q21),add(12)(q24.1),-17,+mar[1]/46,XX,add(10)(q26)[1]/46,XX[17]	25	rAML
**5**	F	57	nAML[1]	M4	31	67	NPM1, NRAS, IDH2, PTPN1	46,XX[20]	16	CR
**6**	M	71	pAML	M5a	62	2.3	ASXL1, IDH2	46,XY[20]	7	DOD
**7**	F	76	pAML	M5	70	22.9	DNMT3A, IDH1, NPM1	46,XX[20]	14	DOD
**8**	F	32	nAML[1]	M1	83	41.1	IDH1, NPM1, GATA2, PTPN11	47,XX,+19[16]/46,XX[4]	13	DOD
**9**	M	80	pAML	N.A	21	1.8	N	47,XY,+8[4]	11	DOD
**10**	M	31	nAML	N.A	91	72.5	IDH2,NPM1, FLT3	46,XY[20]	15	CR
**11**	M	55	nAML[1]	M5b	67	46.9	NPM1,FLT3	46,XY[20]	16	CR
**12**	M	70	rAML	M4	71	50	KIT	46,XY,t(19;19)(q13.2;q13.3)[13]/46,XY[7]	48	DOD
**13**	M	81	pAML	N.A	77	3.7	ASXL1, RUNX1	46,XY[20]	13	DOD
**14**	M	75	nAML[1]	M4	17	97.3	NRAS, DNMT3A	46,XY,inv(3)(q21q26.2)[20]	12	DOD
**15**	M	85	rAML	M4	4	0.8	IDH2	46,XY,t(6;20)(q13;p12)[1]/47,XY,+mar[1]	48	pAML
**16**	F	58	nAML[1]	M4	42	15.8	DNMT3A, KRAS, NPM1,FLT3	46,XX[20]	25	CR

CR, complete remission; DOD, dead of disease; F, female; F/U (m), follow-up (months); M, male; nAML, new untreated AML; pAML, persistent AML; rAML, relapsed AML; WBC, white blood cells
